# Recommendation of Early Surgery in Primary Mitral Regurgitation: Pros and Cons

**DOI:** 10.5935/abc.20160188

**Published:** 2017-01

**Authors:** Levent Cerit

**Affiliations:** Near East University-Nicosia-Cyprus

**Keywords:** Mitral Valve Insufficiency / surgery, Magnetic Resonance Imaging / methods, Echocardiography

**To the Editor,**

I have read the article entitled "Recommendation of Early Surgery in Primary Mitral Regurgitation: Pros and Cons" by Rosa et al.^1^ with great interest, recently published in Arquivos Brasileiros de Cardiologia 2016; 107: 173-5. The investigators reported that currently, the recommendation of mitral surgery for asymptomatic patients is very controversial, since the indication of valvular intervention for symptoms, left ventricular dysfunction and dilatation, recent onset atrial fibrillation or pulmonary arterial hypertension is well consolidated in literature.^1^

Quantifying the mitral regurgitation (MR) in echocardiographic quantification is used primarily to aid grading as mild, moderate, and severe regurgitation. Cardiovascular magnetic resonance (CMR) is able to quantify MR with high accuracy and reproducibility using a combination of left ventricle (LV) volumetric measurements and aortic flow quantification. Myerson et al.^2^ reported that quantifying MR with CMR showed a strong association with the future need for surgery over the subsequent 5 years, demonstrating the potential value of this approach. Previous studies also suggest only moderate agreement between CMR and echocardiography^3,4^ and limited reproducibility for quantitative echocardiographic grading.^5^ Evaluation of MR with CMR showed a significant relation with the future need for mitral valve surgery and was superior to CMR-derived LV volume and echocardiographic grading of regurgitation. These CMR parameters might prove useful for identifying suitable patients for early mitral valve repair replacement.^2^

In the light of these knowledge, CMR parameters might be beneficial to determine suitable patients for early mitral valve repair/replacement.
