# Supplementary material for: Recommendation of Early Surgery in Primary Mitral Regurgitation: Pros and Cons
Source: Arq Bras Cardiol. 2017 Jan;108(1):93–4. doi: 10.5935/abc.20160188 (PMC5245856; doi:10.5935/abc.20160188)
Supplement: Related texts

# Reply

**Authors:** Vitor Emer Egypto Rosa, Flávio Tarasoutchi

**To the Editor,**

Thank you so much for you interest in our Article "Indicação Cirúrgica Precoce na Insuficiência Mitral Primária: Prós e Contras", published in Arquivos Brasileiros de Cardiologia.

Cardiovascular magnetic resonance (CMR) is a method of great value, primarily regarding the anatomical diagnostic of valvular diseases. Its main application, to the present day, occurs in patients in which a clinical and echocardiographic dissociation, namely cases in which propaedeutics indicates an important valvar disease and the echocardiogram describes such injury as moderate, or vice-versa. In these cases, CMR is a better choice instead of cardiac catheterization together with ventriculography, as it seems less invasive and able to quantify more precisely the cardiac chambers volumes and functions.^1^

Myerson et al.^2^ and Enriquez-Sarano et al.^3^ articles show how extremely important is to demonstrate the existence of patient subgroups which could have benefited from an early mitral valvular surgery. Nevertheless, the parameters evaluated in this article need validation in future studies due to the work's pattern under discussion (observational study, non-blinded, surgical recommendation based on the patient's doctor opinion).

Thus, our opinion is that one must pay attention when recommending intervention based solely on the CMR parameters. However, we are in agreement as to such data is able to, in connection with other risk markers (BPF, effective regurgitant orifice, among others), add information for the *Heart Team* clinical decision.

**Dr. Vitor Emer Egypto Rosa****Prof. Dr. Flávio Tarasoutchi**

## References

[1] Tarasoutchi F, Montera MW, Grinberg M, Barbosa MR, Piñeiro DJ, Sánchez CR (2011). Brazilian Guidelines for Valve Disease - SBC 2011 / I
Guideline Inter-American Valve Disease - 2011 SIAC. Arq Bras Cardiol.

[2] Myerson SG, d'Arcy J, Christiansen JP, Dobson LE, Mohiaddin R, Francis JM (2016). Determination of clinical outcome in mitral regurgitation
with cardiovascular magnetic resonance quantification. Circulation.

[3] Enriquez-Sarano M, Avierinos JF, Messika-Zeitoun D, Detaint D, Capps M, Nkomo V (2005). Quantitative determinants of the outcome of asymptomatic
mitral regurgitation. N Engl J Med.
